# Equivalence partition based morphological similarity clustering for large-scale time series

**DOI:** 10.1038/s41598-023-33074-6

**Published:** 2023-04-11

**Authors:** Shaolin Hu

**Affiliations:** Automation School, Guangdong University of Petrochemical Technology, Maoming, 525000 China

**Keywords:** Computational science, Statistics, Chemical engineering

## Abstract

Data clustering belongs to the category of unsupervised learning and plays an important role in the dynamic systems and big data. The clustering problem of sampled time-series data is undoubtedly much more challenging than that of repeatable sampling data. Most of the existing time-series clustering methods stay at the level of algorithm design, lacking rigorous theoretical foundation and being inefficient in dealing with large-scale time series. To address this issue, in this paper, we establish the mathematical theory for the large-scale time series clustering of dynamic system. The main contributions of this paper include proposing the concept of time series morphological isomorphism, proving that translation isomorphism and stretching isomorphism are equivalent relations, developing the calculation method of morphological similarity measure, and establishing a new time series clustering method based on equivalent partition and morphological similarity. These contributions provide a new theoretical foundation and practical method for the clustering of large-scale time series. Simulation results in typical applications verify the validity and practicability of the aforementioned clustering methods.

## Introduction

Large-scale time series clustering is an important tool for simplifying the design of complex equipment process monitoring systems. With the rapid development and wide application of intelligent sensing technology, industrial equipment not only becomes more and more intelligent but also has increasingly complex structures. During the operation of large-scale devices with complex structures, large-scale data sequences that vary over time widely appear and serve as an important basis for sensing equipment status and monitoring equipment failures. Taking the integrated control system of an ethylene plant with a production capacity of 900,000 tons/year as an example, the DCS and SIS systems, as its two core parts, involve as many as 8020 components. The DCS has 9782 I/O communication slots, 97 sets of analytical instruments, 581 flow meters, 246 sets of liquid level instruments, 2965 sets of pressure and temperature instruments, 2319 sets of transmission devices, 1034 control valves, more than 210 sets of air source distributors, and more than 110 km of Tube and Pipe pipelines. During the production process of these equipment and instruments, there are over 100,000 sampling data sequence formed by acquiring data at different sampling times (short as the sampled time series) along with changes in materials, working conditions, and environment. For another example, during the orbit operation of a large spacecraft or constellation, the number of the sampled telemetry time series is typically more than millions. Whether the production process of ethylene or the on-orbit management process of spacecraft, a large number of sampled time series are an important basis for us to monitor production conditions, to detect abnormalities, and to implement safety control, although these sampling sequences are difficult to fully control due to their various modal, non-stationary, and high dynamic changes. For such a large-scale time series, it is very necessary to adopt appropriate clustering methods to cluster the time series to simplify the design complexity and difficulty of monitoring systems.

Time series clustering is a hot research topic in the field of knowledge engineering and machine learning. In the past 20 years, people have conducted a lot of research and proposed a series of clustering and algorithms^1^, which mainly focuses on overall time series clustering, subsequence clustering, and time point clustering, using methods such as distance metric^2,3^, similarity metric^4–6^, spline approximation^7,8^, feature representation^9^, and clustering prototypes^3,10^, etc. In the usual sense, the time series clustering is to partition a large-scale data set of time series into many clusters based on the mutual distance among the data, which means time series data in the same cluster are intrinsically similar, so that time series data in the same cluster are similar. In knowledge discovery and its related fields, there are various methods and algorithms proposed to cluster time series ^11^, including general purpose clustering algorithms commonly used in time series clustering. Besides, existing literatures have considered for evaluating the performance of the clustering performance, and the metrics to determine the resemblance between two time series, either in the forms of raw data, extracted features, or model parameters. Typical practical methods are composed of two steps: the first step is to work out a set of appropriate distance/similarity metrics, and the second step is to build the clustering structures through existing techniques, such as k-means clustering, hierarchical clustering, density based clustering and subspace clustering, etc. Among these, the dynamic time warping (DTW) distance is one of the most commonly adopted metrics for time series clustering. However, the computational complexity of DTW is the main disadvantage that affects its application ^11,12^, especially in the case of handling large volumes of data. Although many techniques including index structure and lower bound function have been proposed to speed up the search, it is remains challenging to search the entire database of the system with limited resources within a given time. More unfavorably, the DTW distance measurement is an algorithm that only measures the similarity of time series in a linear manner ^13^. Although the DTW metric is effective in processing time axis compression and expansion, it is insensitive to amplitude changes ^14^ and does not satisfy the triangular inequality of distance. In order to overcome these drawbacks, Zhou and Hu^15^ proposed an improved morphological weighted DTW algorithm for time series data clustering. Another method is to consider clustering and classification of time series from the perspective of the shape changes ^12,16^. Niennattrakul et al. ^12^ elaborated that the template matching is a solution to efficiently reduce storage and computation load and proposed a template matching framework with using DTW distance, where a shape-based averaging algorithm was utilized to construct significant templates. Besides, they raised a shape-based template matching framework (STMF) to discover a cluster representative of time series and maintain good accuracy against its rivals. Hu et al. ^14^ developed a morphological clustering algorithm based on morphological similarity measurement. Meesrikamolkul et al.^17^ argued that although the k-means similarity clustering with Euclidean distance was one of the most famous algorithms for time series data clustering, its DTW distance makes it impractical because the current averaging functions fail to preserve characteristics of time series within the cluster. Thus, they proposed a shape-based clustering for time series (SCTS) by means of ranking shape-based template matching framework (RSTMF) to average a group of time series effectively. Nevertheless, neither STMF nor SCTS specifically considers the shape of the time series. In particular, most of the time series clustering algorithms are unsupervised machine learning methods. They cannot solve the data clustering problems at the mechanism level, such as determining how many classes can be clustered and why they can be clustered, etc. This makes the clustering methods lack of rigor in mathematical theory. For time series clustering, it is desired that some basic changes (e.g., transverse/longitudinal translation and stretching) do not alter the clustering categories. In other words, some invariance in the time series morphological clustering should be maintained, such as affine invariance, so as to gather the similar time series into the same clustering category.

Due to the significant differences between time series dataset and sampling dataset in mathematical statistics, these time series clustering methods stated above are far from perfect in clustering theory and practical technology. Taking the large-scale time series sampled by a large number of instruments during the operation of ethylene production units aforementioned as an example, sampling time series from different instruments at different stages of time may have different statistical characteristics: strong stationary, weak stationary, and a large number of non-stationary. In order to actually design a process monitoring system based on time series clustering, it is necessary to break through at least three difficult problems which directly related to clustering: (a). Theoretically, there is a need to be able to answer how many categories a large-scale time series dataset should be clustered into; (b). For time series with the same morphology (such as sinusoidal and cosine vibration signals), regardless of their curve length, amplitude, and axis position, they should be clustered into the same cluster; (c) For production processes with periodic characteristics, the change patterns of data in different periods of the instrument are usually similar, and therefore, they cannot be incorrectly clustered in different clusters due to differences in starting time and change intervals. These problems are three unresolved issues that must be addressed in time series clustering research with universal significance. As an important branch of data clustering, although time series clustering similarly inherits the key requirement of "significant differences between different classes and sufficient similarity within each class" derived from data clustering, the characteristics of time series are diverse and multifaceted, how to measure the similarity and evaluate the difference between time series is very complex, and it cannot be separated from the domain of the object, the morphology of time series data, and the application of clustering scenarios. In fact, the aforementioned problems widely exist and limit the rigor of clustering research. Specifically, in the machine learning field, it is generally recognized that clustering is an unsupervised learning process for identifying clusters and that different methods used in cluster analysis often lead to different results. In this way, the number of clusters obtained by different researchers may not be consistent. In order to overcome the above problems, this paper takes time series dataset as the object, and analyzes the clustering problem of large-scale sampling time series, with the aim to establish both a rigorous theoretical foundation and a practical method.

In view of the aforementioned considerations and the practical demand of efficient and rigorous time series clustering, this paper proposes a morphological similarity clustering method based on equivalence relationship. Specifically, in “Methods and main results” section, four groups of equivalence relations and pertaining isomorphism theorems of multi-source time series are established by employing time series sampling data. In “Partitioning large-scale time Series set” section, we develop the equivalent partition theorem of large-scale time series datasets and the partition structure of large-scale datasets. In “Metric of morphological deviation” section, the calculation methods of morphological similarity metrics are proposed, which can be directly used for clustering of time series datasets based on different equivalence relations. In “Two typical application scenarios” section, simulation results of two typical applications are provided, which verify the effectiveness of the proposed clustering methods based on equivalence relations for time series data sets.

## Methods and main results

In order to solve the difficult problem of how many clusters a large-scale time series should be clustered into, which has long plagued the development of clustering theory, the main method proposed in this paper is to adopt the idea of equivalence division, and to propose four kinds of morphological similarity equivalence relationship which are particularly suitable for large-scale time series analysis, as well as four series of morphological similarity measurement algorithms to determine the number of clusters through the morphological similarity equivalence relationship.

The number of clusters is determined by the equivalence relationship of morphological similarity, and the clustering method is determined by the measurement of morphological similarity, which fundamentally advances the establishment and development of clustering theory.

### Analysis of isomorphism and equivalence

The so-called isomorphism of time series is a very important concept in time series clustering. Intuitively, for any two time series, if one of them can be transformed into the other through translation or stretching, we call the two time series isomorphic. In the field of data analysis and statistical learning, isomorphism can be divided into two types: strict isomorphism and weak isomorphism. The former means that one time series can completely coincide with another time series after translation or stretching and the latter means that the mathematical expectation trajectory of one time series can completely coincide with another time series after translation or stretching, Considering that uncertainty error and random disturbance are ubiquitous in the sampling process, this paper focuses on weak isomorphism. In subsequent sections of this paper, the term "isomorphism" refers to “weak isomorphism”. As the time series data transformation, in the mathematical sense, both the translation transformation and the stretching transformation are equivalent relations, which have some important properties such as reflexivity, symmetry and transitivity, etc.

#### Translation isomorphism and equivalence relation

Before specifying that time series are isomorphic with respect to translation, we simplify the description of time series as follows

$$ \{ x_{m} \} = \{ x(t_{m,i} ):\,t_{m,i} = t_{m,0} + (i - 1)h_{m} ,\,i = 1,2,3,...\} $$

where, $t_{m,0}$ is the initial sampling time and $t_{m,i}$ is the *i*-th sampling time and $h_{m}$ is the sampling interval of the *m*-th channel.

##### Definition 1.

For two time series $\{ x_{a} \}$ and $\{ x_{{\text{b}}} \}$, if there is a constant *H* such that the mathematical expectation

1 $$ E\{ x_{a} (t_{a,i} ) - x_{b} (t_{b,i} + H)\} = 0 \quad for\, \forall \;i \in N$$

then $\{ x_{{\text{a}}} \}$ and $\{ x_{{\text{b}}} \}$ are isomorphic with respect to transverse translation; if there is a constant *W* such that

2 $$ E\{ x_{a} (t_{a,i} ) - x_{b} (t_{b,i} )\} = W \quad for\,\forall \;i \in N$$

then $\{ x_{a} \}$ and $\{ x_{b} \}$ are said to be isomorphic with respect to longitudinal translation.

##### Definition 2.

For any two time series $\{ x_{a} \}$ and $\{ x_{b} \}$, whether they are isomorphic for transverse translation, or isomorphic for longitudinal translation, or isomorphic for both transverse translation and longitudinal translation, we call them isomorphic with respect to translation and use $\{ x_{a} \} \approx_{T} \{ x_{{\text{b}}} \}$ to represent the translational isomorphic relationship.

##### Theorem 1

For any two time series $\{ x_{a} \}$ and $\{ x_{{\text{b}}} \}$, $\{ x_{a} \} \approx_{T} \{ x_{{\text{b}}} \}$ if and only if there are two constants *H* and *W* such that

3 $$ E\{ x_{a} (t_{a,i} ) - x_{b} (t_{b,i} + H)\} = W $$

##### Proof:

(a). According to definition 2, if $\{ x_{a} \} \approx_{T} \{ x_{{\text{b}}} \}$, then time times $\{ x_{a} \}$ and $\{ x_{{\text{b}}} \}$ are either transversely translational isomorphic,i.e.

4 $$ \exists H,\;E\{ x_{a} (t_{a,i} ) - x_{b} (t_{b,i} + H)\} = W\quad (W = 0) $$

or longitudinally translational isomorphic, i.e.

5 $$ \exists W,\;E\{ x_{a} (t_{a,i} ) - x_{b} (t_{b,i} + H)\} = W\quad (H = 0) $$

or both transversely translational isomorphic and longitudinally translational isomorphic, i.e.

6 $$ \exists H,W:\;E\{ x_{a} (t_{a,i} ) - x_{b} (t_{b,i} + H)\} = W $$

(b). If there are two constants *H* and *W* such that the formula (3) holds, then, in case (*H* = *0* & *W ≠ 0*), the core trajectories of these two time series can coincide by longitudinally translating *W*; in case (*W* = *0 & H ≠ 0*), the core trajectories of these two time series can coincide by transversely translating *H*; in case ($HW \ne 0$), the core trajectories of these two time series can be coincident by firstly translating *W* longitudinally and then *H* transversely. So, time series $\{ x_{a} \}$ and $\{ x_{{\text{b}}} \}$ are translation isomorphic. ▋

It can be seen from Definition 1 that if $\{ x_{a} \} \approx_{T} \{ x_{{\text{b}}} \}$, then the core trajectories of $\{ x_{a} \}$ and $\{ x_{{\text{b}}} \}$ can be coincident without considering the influence of stochastic disturbance. In other words, translation isomorphic time series can realize the coincidence of core trajectories by the transverse translation as well as the longitudinal translation. Therefore, large-scale time series obtained from multiple different measurement channels can be classified into multiple isomorphic classes through isomorphic reduction or similarity clustering, which can provide great convenience for knowledge extraction and process monitoring.

##### Theorem 2

For large-scale sampled time series, the translation isomorphism $\approx_{{\text{T}}}$ of time series is an equivalent relationship.

##### Proof

The so-called equivalent relation refers to the mathematical relation satisfying reflexivity, symmetry and transitivity. It is easy to verify that the translation isomorphism $\approx_{{\text{T}}}$ satisfies the following three properties:

- *Reflexivity*. In fact, for any time series $$\{ x_{a} \}$$, the relation $$E\{ x_{a} (t_{a,i} ) - x_{a} (t_{a,i} )\} = 0$$ holds, so we have $$\{ x_{a} \}$$$$\approx_{{\text{T}}}$$$$\{ x_{a} \}$$;
- *Symmetry*. In fact, if the constants $(H,W)$ exists so that $\{ x_{a} \} \approx_{T} \{ x_{{\text{b}}} \}$ holds, then $\tilde{H} = - H$ and $\tilde{W} = - W$ can be selected so that $E\{ x_{{\text{b}}} (t_{b,i} ) - x_{a} (t_{a,i} + \tilde{H})\} = \tilde{W}$ holds, and thus the relationship $\{ x_{{\text{b}}} \} \approx_{T} \{ x_{{\text{a}}} \}$ holds;
- *Transitivity*. In fact, if $\{ x_{a} \} \approx_{T} \{ x_{b} \}$ and $\{ x_{b} \} \approx_{T} \{ x_{c} \}$ with constants $\{ H,W\}$ and $\{ H^{\prime},W^{\prime}\}$ respectively, setting constants $\tilde{H} = H + H^{\prime}$ and $\tilde{W} = W + W^{\prime}$, we get $E\{ x_{a} (t_{a,i} ) - x_{{\text{c}}} (t_{c,i} + \tilde{H})\} = \tilde{W}$, and $\{ x_{a} \} \approx_{T} \{ x_{c} \}$.

The above properties show that translational isomorphism satisfies the three properties of reflexivity, symmetry and transitivity, and is an equivalence relation.

#### Stretching isomorphism and equivalence relation

The stretching isomorphism of the shape of time series graph is another important isomorphism of time series, which means that one time series can overlap with another time series graph by elongating or compressing the scatter graph. The stretching isomorphism includes transversely stretching isomorphism and longitudinally stretching isomorphism.

##### Definition 3.

For two time series $\{ x_{a} \}$ and $\{ x_{b} \}$, if the following formula (7) holds:

7 $$ E\{ x_{a} {(}t_{a,1} + {(}i - 1{)}h_{a} {)} - x_{b} {(}t_{b,1} + {(}i - 1{)}h_{b} {{)\} }} = 0 \quad for\, \forall \;i \in N $$

where $h_{{\text{a}}}$ and $h_{b}$ are the sampling intervals of $\{ x_{a} \}$ and $\{ x_{b} \}$ respectively, then these two time series $\{ x_{a} \}$ and $\{ x_{b} \}$ are isomorphic with respect to stretching time interval; if there exists a constant *C* such that

8 $$ E\{ x_{a} (t_{a,i} ) - Cx_{b} (t_{b,i} )\} = 0 \quad for\, \forall \;i \in N $$

then these two time series $\{ x_{a} \}$ and $\{ x_{{\text{b}}} \}$ are isomorphic with respect to stretching range. ▋

For any two time series $\{ x_{a} \}$ and $\{ x_{b} \}$, if they are transverse stretching isomorphic, longitudinal stretching isomorphic, or both, they are referred to as stretching isomorphic and the relation of these two time series $\{ x_{a} \}$ and $\{ x_{b} \}$ are denoted by $\{ x_{a} \} \approx_{S} \{ x_{b} \}$.

It can be seen from Definition 3 that if $\{ x_{a} \} \approx_{S} \{ x_{b} \}$, then the core trajectories of $\{ x_{a} \}$ and $\{ x_{b} \}$ can be coincident without considering the influence of stochastic disturbance. In other words, two stretching isomorphic time series can realize the coincidence of core trajectories by the transversely stretching as well as the longitudinally stretching.

##### Theorem 3

For any two time series $\{ x_{a} \}$ and $\{ x_{b} \}$, $\{ x_{a} \} \approx_{S} \{ x_{b} \}$ if and only if there are two constants *C* and *D* such that

9 $$  E\{ x_{a} (t_{a0} + ih_{a} ) - Cx_{b} (t_{b,0} + ih_{b} D)\} = 0 \quad for\, \forall \;i \in N $$

##### Proof:

Given the proof process of Theorem 1 and combining with Definition 3, it follows directly that this theorem holds.

##### Theorem 4

For large-scale time series, the stretching isomorphism relation $\approx_{{\text{S}}}$ is an equivalent relationship.

##### Proof

In order to deduce that the relation $\approx_{S}$ is equivalent, we only need to prove that the relation $\approx_{S}$ satisfies reflexivity, symmetry and transitivity.

- *Reflexivity*. In fact, for any sampling time series $\{ x_{a} \}$, $E\{ x_{a} (t_{a,i} ) - x_{a} (t_{a,i} )\} = 0$ holds, and $\{ x_{a} \} \approx_{S} \{ x_{a} \}$ is true.
- *Symmetry*. In fact, if $\{ x_{a} \} \approx_{S} \{ x_{b} \}$ with constants *C* and *D*, then the following expression is hold
  10 $$ E\{ x_{a} {(}t_{a,0} + ih_{a} {)} - Cx_{b} {(}t_{b,0} + ih_{b} D)\} = 0  \quad for\,forall \;i \in N $$

Selecting $\tilde{C} = C^{ - 1}$ and $\tilde{D} = D^{ - 1}$, we have

11 $$ E\{ x_{b} {(}t_{b,0} + ih_{b} {)} - \tilde{C}x_{a} {(}t_{a,0} + ih_{a} \tilde{D}{{)\} }} = 0 \quad for\, \forall \;i \in N $$

Therefore, $\{ x_{{\text{b}}} \} \approx_{{\text{S}}} \{ x_{{\text{a}}} \}$ is true.

c) *Transitivity*. In fact, if $\{ x_{a} \} \approx_{S} \{ x_{b} \}$ and $\{ x_{b} \} \approx_{S} \{ x_{{\text{c}}} \}$ with constants {*C,D*} and {*C’,D’*} respectively, then we have the following mathematical expressions:
  12 $$ E\{ x_{b} {(}t_{b,0} + ih_{b} {)} = \frac{1}{C}Ex_{a} {(}t_{a,0} + ih_{a} \frac{1}{D})\} \,\, E\{ x_{c} {(}t_{c,0} + ih_{c} {)} = \frac{1}{C\,^{\prime}}Ex_{b} {(}t_{b,0} + ih_{\,b} \frac{1}{D\,^{\prime}})\} \quad for\,\, \forall \;i \in N $$

Selecting constants $\tilde{C} = CC^{\prime}$ and $\tilde{D} = DD^{\prime}$, the following expression (14) can be derived by formula (12) and (13), i.e.

13 $$ E\{ x_{c} {(}t_{c,0} + ih_{\,c} {)} = \frac{1}{{\tilde{C}}}Ex_{a} {(}t_{a,0} + ih_{\,a} \frac{1}{{\tilde{D}}})\} \quad for\, \forall \;i \in N$$

So, $\{ x_{a} \} \approx_{S} \{ x_{c} \}$ is true, and the transitivity holds. ▋

The translation equivalence relation $\approx_{T}$ and the stretching equivalence relations $\approx_{S}$ stated above are very useful for partitioning, clustering and classifying large-scale time series.

### Partitioning large-scale time series set

The partition theorem based on the equivalence relation in set theory^18,19^ shows that any set can be uniquely and definitely divided into several equivalence subsets by any equivalence relation defined on the set, satisfying that different elements in each class are equivalent. Therefore, it is possible to use the translation isomorphism given in Theorem 2 and the stretching isomorphism given in Theorem 4 to divide a large-scale time series set into several equivalent class subsets.

Without loss of generality, use the set *A* to represent the multi-source sampling time series set obtained by the multi-sensor monitoring network composed of *M* sensors, which can be expressed in the following form:

14 $$ A = \{ \{ x_{a} \} |x_{a} (t_{a,i} ) \in R;a = 1,...,M;i = 1,2,...\} \} $$

and set up equivalence relation $R \in \{ \approx_{T} , \approx_{S} \}$ on set *A*. Then the following Theorem 5 can be derived directly from the set partition theory^18^ based on equivalence relation.

#### Theorem 5

Given that $A \ne \Phi$ and the equivalent class is denoted as $[x_{a} ]_{R} = {{\{ \{ }}x_{b} \} |\{ x_{b} \} \in A,\{ x_{b} \} R\{ x_{a} \} \}$, the following properties hold: (a) the equivalent class $[x_{a} ]_{R} \ne \Phi$ for any series $\{ x_{a} \} \in A$; (b) if $\{ x_{a} \} \in A$ and $\{ x_{b} \} \in A$ are isomorphic, then $[x_{a} ]_{R} = [x_{b} ]_{R}$; (c) if two series $\{ x_{a} \} \in A$ and $\{ x_{b} \} \in A$ are not isomorphic with respect to $R \in \{ \approx_{T} , \approx_{S} \}$, then $[x_{a} ]_{R} \cap [x_{b} ]_{R} = \Phi$; (d) for any time series $\{ x_{c} \} \in A$, it belongs to and only belongs to an equivalent cluster; (e) The union of all clusters is equal to the complete set, i.e. $\cup_{{\{ x_{a} \} }} [x_{a} ] = A$.

#### Proof

Firstly, for the equivalence relation $R \in \{ \approx_{T} , \approx_{S} \}$, and the time seires $\{ x_{a} \} \in A$, the following Eq. (15) holds

15 $$ E\{ x_{a} (t_{a,i} ) - x_{a} (t_{a,i} )\} = 0 $$

So we have

16 $$ \{ x_{a} \} \in [x_{a} ]_{R}  \Rightarrow  [x_{a} ]_{R} \ne \Phi $$

Secondly, for any two time series $\{ x_{a} \} \in A$ and $\{ x_{b} \} \in A$, if the intersection set of equivalent calsses $[x_{a} ]_{R}$ and $[x_{b} ]_{R}$ is not empty set, $[x_{a} ]_{R} \cap [x_{b} ]_{R} \ne \Phi$, then there is a time series $\{ y(t_{i} )\} \in [x_{a} ]_{R} \cap [x_{b} ]_{R}$ satisfying the following equivalent realtion

17 $$ \{ y(t_{i} )\} \approx_{R} \{ x_{a} \} \quad \{ y(t_{i} )\} \approx_{R} \{ x_{b} \}$$

Using the transitive properties of equivalence relation given in Theorem 2 and Theorem 4, we get

18 $$ \{ x_{a} \} \approx_{R} \{ y(t_{i} )\} \approx_{R} \{ x_{b} \} $$

which means that these two series $\{ x_{a} \} \in A$ and $\{ x_{b} \} \in A$ are isomorphic with respect to R. Further, if $\{ x_{a} \} \in A$ and $\{ x_{b} \} \in A$ are isomorphic, we have the following equivalent relation (19) for any time series $\{ z(t_{i} )\} \in [x_{a} ]_{R}$

19 $$ \{ z(t_{i} )\} \approx_{R} \{ x_{a} \} \,\, \{ z(t_{i} )\} \approx_{R} \{ x_{b} \} $$

Using the transitive properties of equivalence relation given in Theorem 2 and Theorem 4, the relation (20) is holds

20 $$ \{ z(t_{i} )\} \approx_{R} \{ x_{b} \}  \Rightarrow  [x_{a} ]_{R} \subseteq [x_{b} ]_{R} $$

Similarly, using the transitive properties of equivalence relation given in Theorem 2 and Theorem 4, the relation (21) is holds

21 $$ [x_{b} ]_{R} \subseteq [x_{a} ]_{R} $$

So, we get $[x_{a} ]_{R} = [x_{b} ]_{R}$. Using the Theorem 5(b), it can be deduced that Theorem 5(c)-(e) are valid. ▋

Theorem 5 indicates us that a group of large-scale time series can be divided into several non-intersecting subsets, by means of stretching isomorphism $\approx_{S}$ or translation isomorphism $\approx_{T}$. Moreover, all of the different time series from the same cluster are isomorphic, and any two time series from two different separate subsets are not isomorphic. These important features should undoubtedly become the mathematical mechanism of large-scale time series clustering.

Theorem 5 is significant as it makes up for the incomplete-ness of existing time series clustering algorithms by providing reasonable mathematical explanation. The innovation of Theorem 5 lies in that it solves the technical problem of how many clusters are most suitable for a given large-scale time series set by introducing the idea of equivalent relationship clustering. Specifically, Theorem 5 shows that for large-scale time series, the number of the most suitable clustering classes is equal to the number of quotient sets,i.e.,

22 $$ A{/}R = \{ [x_{a} ]_{R} |\{ x_{a} \} \in A\} $$

According to the theory of quotient set^19^, any equivalence relation on set *A* can uniquely determine a clustering partition of set *A*. Therefore, if we use the above two equivalence relations $R \in \{ \approx_{T} , \approx_{S} \}$, the large-scale time series set *A* can be divided into four parts. Any two time series in Part 1 are both translation isomorphic and stretching isomorphic, any two time series in Part 2 are translation isomorphic but not stretching isomorphic, any two time series in Part 3 are stretching isomorphic but not translation isomorphic, and any two time series in Part 4 are neither translation isomorphic nor stretching isomorphic. Each partition is a set of subsets of several equivalent classes.

### Metric of morphological deviation

Before using these theoretical results to the actual clustering calculation and knowledge acquisition, it is necessary to give a calculation method for the morphological similarity of time series from the perspective of computational mathematics.

For any two time series, their similarity is inversely proportional to their deviation degree, that is, we can start from the measurement of deviation to establish the similarity measurement index. In this section, four indictors are built to measure deviation and used to measure the morphological similarity of different time series data.

- Metric of minimum deviation under transverse translation

According to Definition 1 and Eq. (1), the minimum deviation $T_{T,a \to b}$ of two time series $\{ x_{a} \}$ and $\{ x_{b} \}$ under transverse translation can be theoretically expressed by the mathematical expectation of absolute deviation, i.e.,

23 $$ T_{T,a \to b} = \mathop {{\text{minizing}}}\limits_{H \in R} E\left| {x_{a} (t) - x_{b} (t + H)} \right| $$

Obviously, $T_{T,a \to b}$ is suitable to measure the difference between time series $\{ x_{a} \}$ and $\{ x_{b} \}$ under transverse translation and can be estimated as

24 $$ \hat{T}_{T,a \to b} = \mathop {{\text{minizing}}}\limits_{H \in R} \sqrt {\frac{1}{N}\sum\limits_{i = 1}^{N} {(x_{a} (t_{i} ) - x_{b} (t_{i} + H))^{2} } } $$

In order to determine the minimization involved in (24), a two-step algorithm is proposed as follows:

*Step 1*: Determine number of sliding steps $m$ by solving the minimum point in the following formula (25)

25 $$ \tilde{m}_{b} = \mathop {{\text{arg}}}\limits_{{}} \mathop {\text{minizing }}\limits_{m \in J} \sum\limits_{i = 1}^{N} {(x_{a} (t_{a,i} ) - x_{b} (t_{b,i} + mh_{b} ))^{2} } $$

where *J* is the integer set and $h_{b}$ is the sampling interval of the time series $\{ x_{{b,{\text{i}}}} \}$.

*Step 2*: Determine the remnants $\delta_{b}$ of time by solving the minimum point in the following formula (26)

26 $$ \hat{\delta }_{b} = \mathop {{\text{arg}}}\limits_{{}} \mathop {{\text{minizing}}}\limits_{{d_{b} \in [ - h_{b} ,h_{b} ]}} \sum\limits_{i = 1}^{N} {(x_{a} (t_{a,i} ) - x_{b} (t_{b,i} + \tilde{m}_{b} h_{b} + \delta_{b} ))^{2} } $$

Then, the minimum deviation metric $T_{T,a \to b}$ between time series $\{ x_{a} \}$ and $\{ x_{b} \}$ under transverse translation can be estimated by

27 $$ \hat{T}_{T,a \to b} = \frac{1}{\sqrt N }\sqrt {\sum\limits_{i = 1}^{N} {(x_{a} (t_{i} ) - x_{b} (t_{i} + \tilde{m}_{b} h_{b} + \hat{\delta }_{b} ))^{2} } } $$

(b) Metric of Minimum Deviation under Longitudinal Translation

According to Definition 1 and Eq. (2), the minimum deviation metric $T_{L,a \to b}$ of any two time series $\{ x_{a} \}$ and $\{ x_{b} \}$ under longitudinal translation is estimated by.

28 $$  T_{L,a \to b} = \mathop {{\text{minizing}}}\limits_{W \in R} E\left| {x_{a} (t) - (x_{b} (t + W)} \right| $$

which can be used to describe the difference between time series $\{ x_{a} \}$ and $\{ x_{b} \}$ with respect to the longitudinal translation. Then, the minimum deviation metric $T_{L,a \to b}$ with respect to longitudinal translation can be estimated by

29 $$ \hat{T}_{L,a \to b} = \frac{1}{\sqrt N }\sqrt {\mathop {{\text{minizing}}}\limits_{W \in R} \sum\limits_{i = 1}^{N} {(x_{a} (t_{i} ) - x_{b} (t_{i} ) - W} )^{2} } $$

Using $\overline{x}_{a}$ and $\overline{x}_{b}$ to represent the statistical mean estimators of $\{ x_{a} (t)\}$ and $\{ x_{b} (t)\}$ respectively, we are able to verify that the following formula (30) is true:

30 $$ \hat{W} = \mathop {\arg {\text{minizing}}}\limits_{W \in R} \sum\limits_{i = 1}^{N} {(x_{a} (t_{i} ) - x_{b} (t_{i} ) - W)^{2} } = \frac{1}{N}\sum\limits_{i = 1}^{N} {(x_{a} (t_{i} ) - x_{b} (t_{i} ))} = \overline{x}_{a} - \overline{x}_{b} $$

According to Eq. (30), $\hat{W}$ is called the least deviation offset. The residual sequence $\{ E_{a,b} (t_{i} )\}$ under the longitudinal translation based on the least deviation is as follows

31 $$  E_{a,b} (t_{i} ) = x_{a} (t_{i} ) - (x_{b} (t_{i} ) - \hat{W}) = [x_{a} (t_{i} ) - \overline{x}_{a} ] - [x_{b} (t_{i} ) - \overline{x}_{b} ] $$

It can be seen that calculation formula (29) of the minimum deviation under the longitudinal translation can be estimated by

32 $$ \hat{T}_{L,a \to b} = \sqrt {\frac{1}{N}\sum\limits_{i = 1}^{N} {E_{a,b}^{2} (t_{i} )} } $$

(c) Metric of Minimum Deviation under Transverse Stretching

Based on Definition 3 and Eq. (7), the minimum deviation $S_{T,a \to b}$ of any two time series $\{ x_{a} \}$ and $\{ x_{b} \}$ under transverse stretching is defined by

33 $$ S_{T,a \to b} = \mathop {{\text{minizing}}}\limits_{D \in [0, + \infty )} E|x_{a} (t) - x_{b} (t_{0} + D(t - t_{0} ))| $$

which can be used to describe the difference between time series $\{ x_{a} \}$ and $\{ x_{b} \}$ with respect to the transverse stretching. Then, the minimum deviation metric $S_{T,a \to b}$ under transverse stretching can be estimated by the formula (34)

34 $$ \hat{S}_{T,a \to b} = \sqrt {\frac{{1}}{N}\mathop {{\text{minizing}}}\limits_{D \in [0, + \infty )} \sum\limits_{i = 1}^{N} {(x_{a} (t_{a,0} + ih_{a} ) - x_{b} (t_{b,0} + ih_{b} D))^{2} } } $$

In order to determine the minimization of the formulae (34), a two-step algorithm is proposed as follows:

*Step 1*: According to the *First Weierstrass Approximation Theorem*, the sampling time series can be fitted with *k*-order polynomials, where the value of *k* can be determined by *Akachi Information Criterion*. Using the classical least squared fitting method, we can construct the vectors formed by the fitting coefficients

35 $$ \left( {\begin{array}{*{20}c} {\hat{\theta }_{z,1} } \\ \vdots \\ {\hat{\theta }_{z,kz} } \\ \end{array} } \right) = \left( {H_{z,k} } \right)^{ - 1} \left( {\begin{array}{*{20}c} {t_{z,1}^{0} } & \cdots & {t_{z,1}^{k} } \\ \vdots & {} & \vdots \\ {t_{z,N}^{0} } & \cdots & {t_{z,N}^{k} } \\ \end{array} } \right)^{\tau } \left( {\begin{array}{*{20}c} {x_{z} (t_{z,1} )} \\ \vdots \\ {x_{z} (t_{z,N} )} \\ \end{array} } \right)\quad (z = a,b) $$

and the fitting curves $\hat{f}_{a} (t)$ and $\hat{f}_{b} (t)$ are as follows

36 $$ \hat{f}_{z} (t) = \sum\limits_{i = 0}^{{k_{z} }} {\hat{\theta }_{z,i} t^{i} } \quad (z = a,b) $$

for $\{ x_{a} \}$ and $\{ x_{b} \}$ respectively, where the matrices $H_{z,k}$ are determined by the following formula

37 $$ H_{z,k} = \left( {\begin{array}{*{20}c} {t_{z,1}^{0} } & \cdots & {t_{z,1}^{k} } \\ \vdots & {} & \vdots \\ {t_{z,N}^{0} } & \cdots & {t_{z,N}^{k} } \\ \end{array} } \right)^{\tau } \left( {\begin{array}{*{20}c} {t_{z,1}^{0} } & \cdots & {t_{z,1}^{k} } \\ \vdots & {} & \vdots \\ {t_{z,N}^{0} } & \cdots & {t_{z,N}^{k} } \\ \end{array} } \right)\quad (z = a,b) $$

*Step 2*: Determine the transverse stretching ratio *D* by

38 $$ \hat{D} = \arg \mathop {{\text{minizing}}}\limits_{D \in [0, + \infty )} \int_{{t_{a,1} }}^{{t_{a,N} }} {\left( {\hat{f}_{a} (t) - \hat{f}_{b} (t_{a,1} + D(t - t_{a,1} ))} \right)}^{2} dt $$

and calculate the difference metric under transverse stretching by the following formula

39 $$ \hat{S}_{T,a \to b} = \int_{{t_{a,1} }}^{{t_{a,N} }} {\left( {\hat{f}_{a} (t) - \hat{f}_{b} (t_{a,1} + \hat{D}(t - t_{a,1} ))} \right)}^{2} dt $$

(d) Metric of Minimum Deviation under Longitudinal Stretching

According to Definition 3 and Eq. (8), the minimum deviation $S_{L,a \to b}$ of any two time series $\{ x_{a} \}$ and $\{ x_{b} \}$ under longitudinal stretching is defined by

40 $$ S_{L,a \to b} = \mathop {{\text{minizing}}}\limits_{C \in (0, + \infty )} \sqrt {E(x_{a} (t) - Cx_{b} (t))^{2} } $$

which can be used to describe the difference between time series $\{ x_{a} \}$ and $\{ x_{b} \}$ with respect to the longitudinal stretching. Then, the minimum deviation metric $S_{L,a \to b}$ under longitudinal stretching is constructed as follows

41 $$ \hat{S}_{L,a \to b} = \frac{1}{\sqrt N }\mathop {{\text{minizing}}}\limits_{C \in (0, + \infty )} \sqrt {\sum\limits_{{{\text{i}} = {1}}}^{{\text{N}}} {(x_{a} (t_{a,i} ) - Cx_{b} (t_{b,i} ))^{2} } } $$

It is easy to verify that the following formula (42) is true,

42 $$ \hat{C} = \frac{{1}}{N}\mathop {\arg \min {\text{izing}}}\limits_{C \in R} \sum\limits_{i = 1}^{N} {(x_{a} (t_{a,i} ) - Cx_{b} (t_{b,i} ))^{2} } = \frac{{\sum\limits_{i = 1}^{N} {x_{a} (t_{a,i} )x_{b} (t_{b,i} )} }}{{\sum\limits_{i = 1}^{N} {x_{b} (t_{b,i} )x_{b} (t_{b,i} )} }} $$

For two time series $\{ x_{a} \}$ and $\{ x_{b} \}$, selecting $\hat{C}$ as the optimal elongation coefficient and adopting the notation

43 $$ R_{a,b} = \frac{1}{N}\sum\limits_{i = 1}^{N} {x_{a} (t_{a,i} )x_{b} (t_{b,i} )} $$

then the residual sequence $\{ F_{a,b} (t_{i} )\}$ under the longitudinal expansion based on the optimal elongation is as follows

44 $$ F_{a,b} (t_{a,i} ) = x_{a} (t_{a,i} ) - \frac{{R_{a,b} }}{{R_{b,b} }}x_{b} (t_{b,i} ) $$

and the estimator of $S_{L,a \to b}$ can be transformed into

45 $$ \hat{S}_{L,a \to b} = \frac{1}{\sqrt N }\sqrt {\sum\limits_{{{\text{i}} = {1}}}^{{\text{N}}} {F_{a,b}^{2} (t_{a,i} )} } $$

It is worth noting that these aforementioned metrics unifies the conventional time series clustering methods based on Euclidean distance, the Shape-based template matching, and the Dynamic Time Warping (DTW).

Moreover, when using the above minimum deviation metrics for machine learning and clustering of time series data, the errors in the time series of measurement data must be considered. This is because of the inevitable measurement errors and random disturbances. In other words, it is difficult to obtain a zero-value metric of the minimum deviation when morphological clustering is carried out on the actual sampled data. Nevertheless, the closer the minimum deviation metric is to zero, the higher the similarity is.

## Two typical application scenarios

These proposed methods of equivalent division based on morphological similarity and clustering based on similarity metric are very valuable in many different fields such as clustering of massive time series, monitoring of on-orbit spacecraft operation status, monitoring of petrochemical production process, monitoring of process abnormal changes, etc. In this section, two typical applications are provided, where the proposed methods are used for reduction of massive monitoring data and for detection of abnormal changes in times series, respectively.

### Scenario 1: reduction of massive monitoring data

Reduction of large-scale sampled data is an important issue in the field of process monitoring for complex objects. In order to monitor the dynamic changes of complex dynamic objects, it is often necessary to use large-scale sensor networks to obtain time series data of dynamic objects from different perspectives or levels. It is difficult and bandwidth-consuming to transmit a large amount of sampling data to the monitoring center. To do this, we need to reduce the large amount of redundant data. In other words, For actual project status monitoring, if there are multiple sets of data sequences with similar changes, we only need to transmit a set of time series data to the monitoring center, thereby reducing a large number of redundant data sequences. A feasible and practical method is to cluster tens of thousands or even hundreds of thousands of sampling time series from sensors. Although the sampling objects of multi-position sensors may be different in the actual engineering process, the change patterns of time series often have some similarity. This feature provides the possibility for clustering-based analysis and diagnosis.

**Figure 1:**
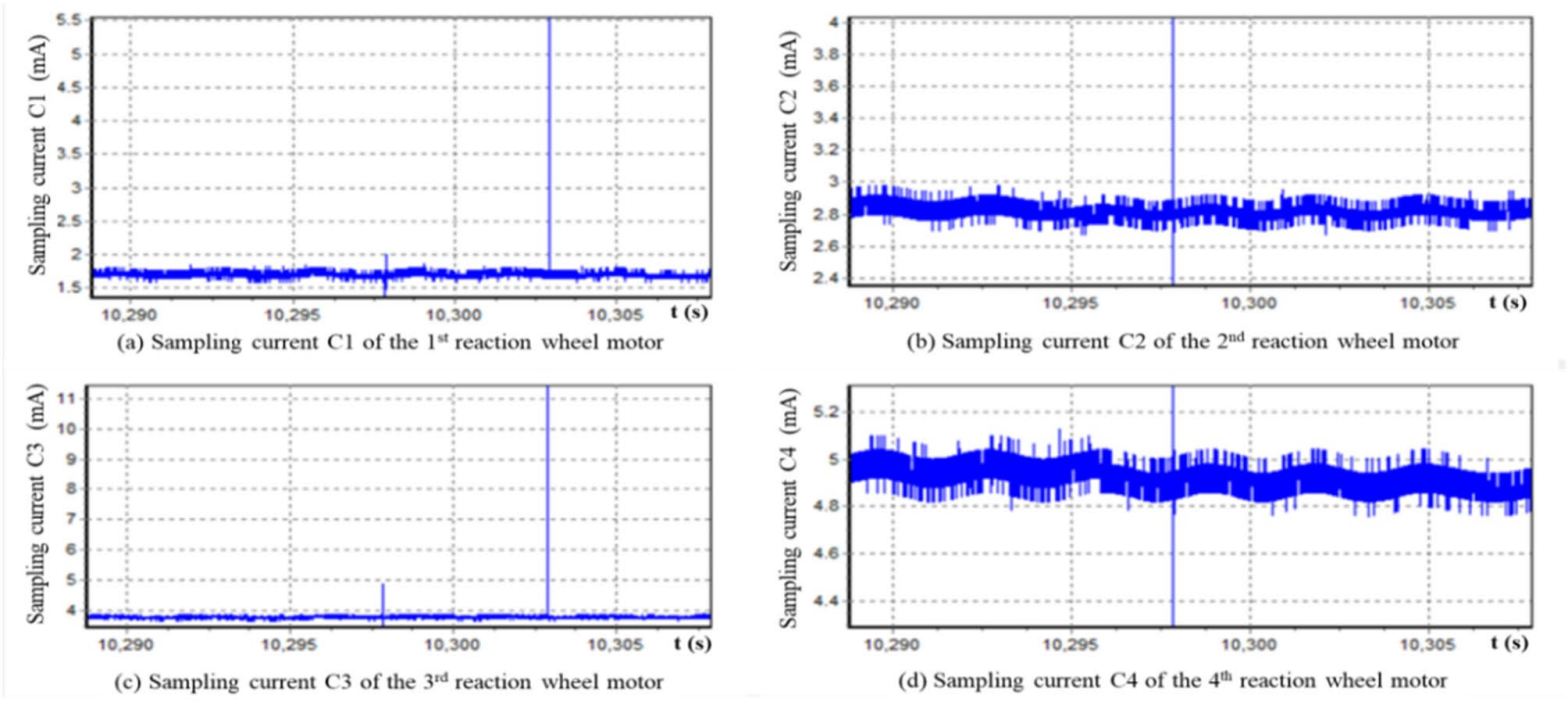
Plot of sampling current data from four reaction wheel motors of an on-orbit spacecraft.

For example, as one of the many functional systems of a satellite, the attitude control system is usually equipped with four reaction wheels. Each reaction flywheel typically has more than 10 different types of sampled data. The current sampling data is directly used to monitor whether the reaction flywheel is working properly. If it can be determined that the current change patterns of the four reaction flywheels are the same, we may significantly reduce the number of current telemetry data downloads, and reasonably use the difference between data changes to find possible faulty flywheels. Figure 1 is the sampling data plots of motor currents of four reaction wheels during spacecraft in orbit.

Using the sampled current data from the four series shown in Fig. 1, it can be seen that the amplitudes of the four series of data are significantly different, but their morphology is somewhat similar. We need to determine whether these four sets of data are essentially homogeneous. If they are isomorphic, we may focus our attention from four sets of data to one set of data when monitoring the flywheel current, thereby effectively reducing the workload.

For this reason, we use Eq. (32) to calculate the minimum deviation for each two groups of these four time series. The results are shown in the following matrix (46), which indicates that all minimum deviations between any two time series in the four time series are very close to zero (it is worth noting that the closer the estimated minimum deviation is to zero, the more similar the two time series are).46

**Figure 2:**
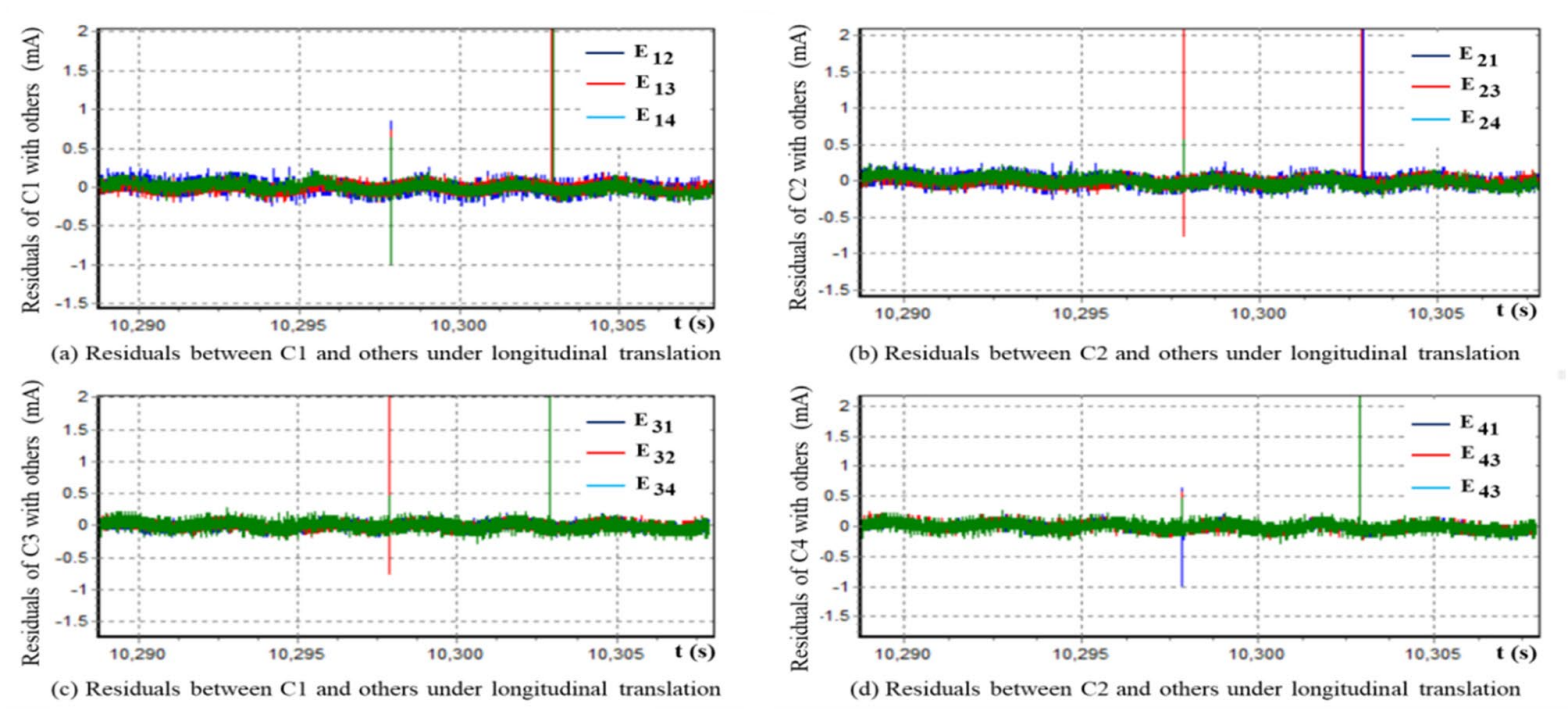
Residuals of sampling current of four reaction wheel motors under longitudinal translation.

Figure 2 is the plots of the residuals calculated according to formula (31) under the longitudinal translation equivalent transformation, which can be observed that the four groups of data are longitudinally translational equivalent and thus belong to the same morphological isomorphic equivalent cluster. Further, it can be concluded that the four different sampling time series shown in Fig. 2 are almost identical statistically.

The metric matrix TL of the minimum deviation under longitudinal translation conditions shown in Eq. (46) calculated using measured data shows that the morphological differences of the four time series can be eliminated through appropriate longitudinal translation, which is also confirmed by Fig. 2. Therefore, when the flywheel operates normally, the workload of simultaneously monitoring the currents of four flywheels can be simplified to monitoring the current data of one flywheel. If some flywheels may be abnormal, we can use the morphological similarity of the four modes under normal conditions to determine whether the flywheel data maintains a similar morphology, thereby locating the faulty flywheel.

### Scenario2: detection of abnormal changes in time series

The aforementioned morphological similarity metrics can not only cluster time series data with similar morphology, but also distinguish time series with different morphology, thereby detecting inconsistencies and abnormal changes. Taking a heating furnace as an example, we can use the method of monitoring the morphological differences of petrochemical production process sampling data obtained from a large number of sensors and instruments during the petrochemical production process to achieve the monitoring of production equipment status changes and implement safe operation management.

**Figure 3:**
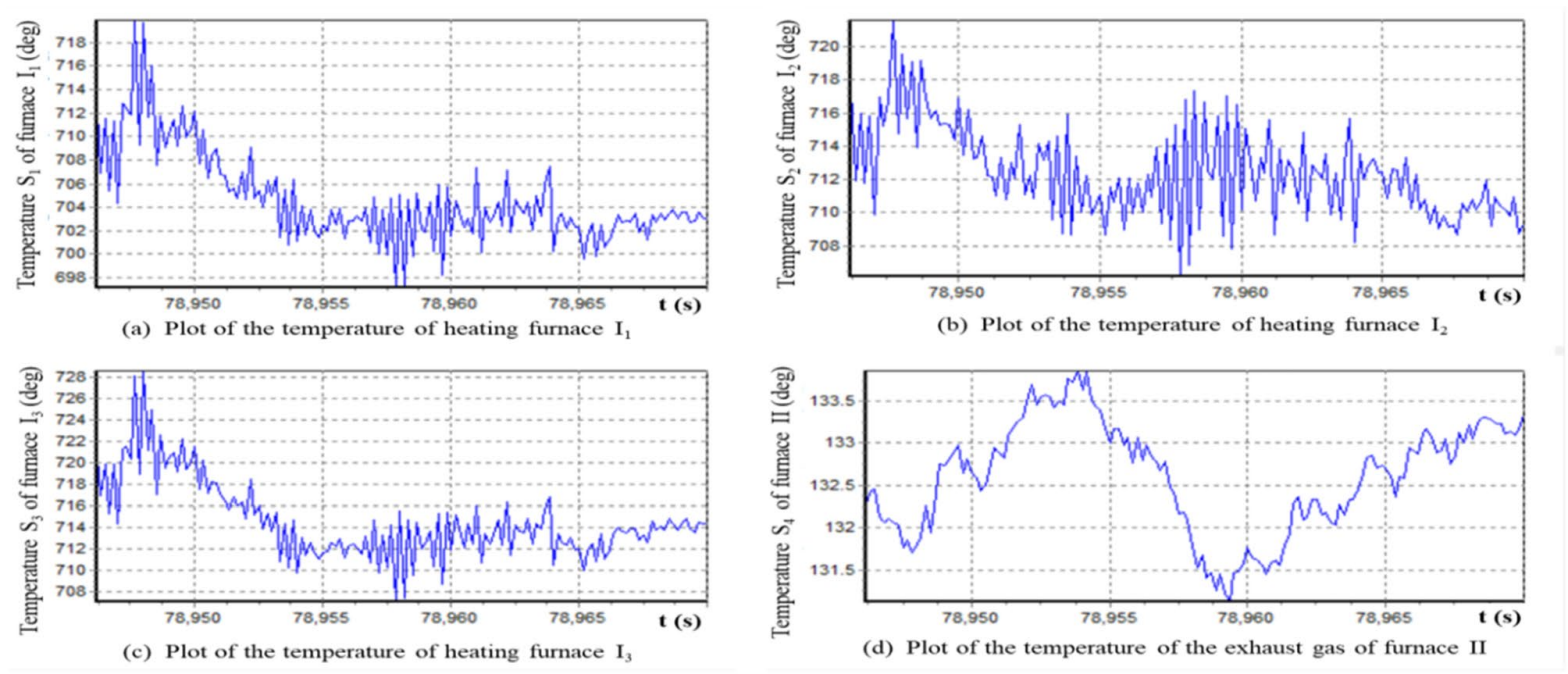
Plots of the temperature of furnace I and the temperature of the exhaust gas from furnace II.

Heating furnaces are important equipment in petrochemical industry, and their temperature changes are the main basis for monitoring the working conditions of heating furnaces. Whether the temperature changes at different measuring points of the heating furnace are similar is of great significance for simplifying temperature monitoring and finding corrosion and damage in the heating furnace. Figures 3(a-c) show the temperature curves at the measuring points I_1_, I_2_ and I_3_ of the heating furnace I seperately. Besides, Fig. 3(d) shows the exhaust gas temperature curve of the heating furnace II. In the followings, we find out which data sequence has different change pattern compared with the others from the four groups of data.

**Figure 4:**
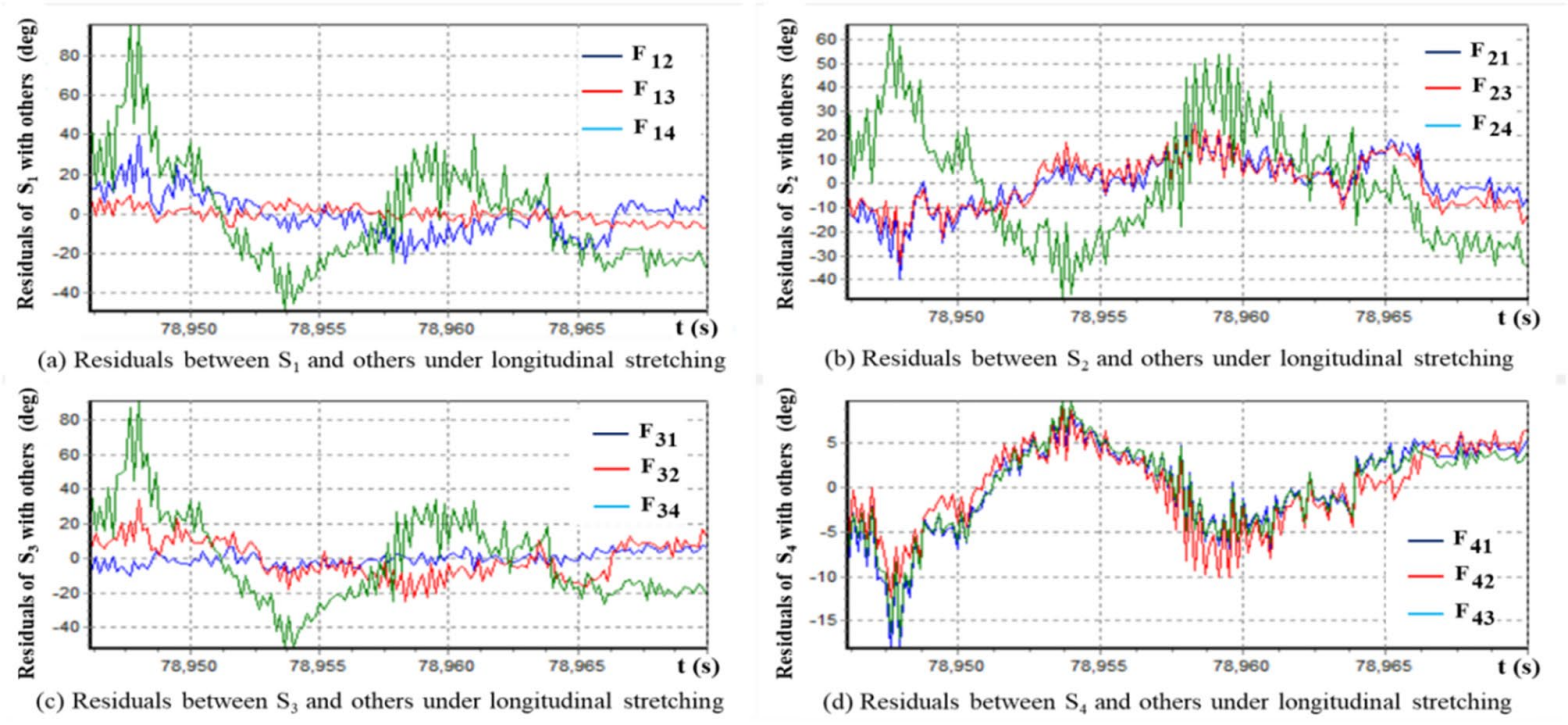
Plot of residual temperature difference based on longitudinal stretching transformation.

According to formula (44), we calculate the residual of the variation under the longitudinal expansion based on the optimal elongation, as shown in Fig. 4. It can be observed that these four sets of data are neither longitudinally stretched equivalent nor belong to the same morphological isomorphic equivalent cluster. Furthermore, it can be concluded that the change patterns of sequences S_2_ and S_4_ are different from the cluster {S_1_, S_3_}, and there are abnormal changes in the changing pattern of time series.

Intuitively, Fig. 4a,c show that the pattern of time series S_1_ is similar to that of S_3_. Figure 4b shows that the pattern of S_2_ is significantly different from those of the other three time series. Figure 4d shows that the pattern of S_4_ is significantly different from those of the other three time series. The question now is how to confirm the above inconsistency and whether the changing patterns of time series S_1_, S_2_, and S_3_ are inconsistent.

In order to rigorously measure the morphological differences and similarities between the aforementioned four time series, we use the morphological similarity metric formulae (45) to estimate the longitudinal stretching similarity metrics between any two radiation time series of the four monitoring points. Results are obtained as follows47

It can be clearly seen from the similarity metric matrix that time series S_1_ and S_3_ are similar in morphology, but S_2_ and S_4_ are not morphological similarity with S_1_ and S_3_. In other words, these four time series are morphologically divided into three categories, i.e., {S_1_, S_3_}, {S_2_}, and {S_4_}. It can also be confirmed from Fig. 4 that it is appropriate to divide these four groups of time series into three clusters.

## Discussion

This study proposes the concept of time series morphological isomorphism, proves that translation isomorphism and stretch isomorphism are equivalent relationships, develops computational methods for morphological similarity measures, and establishes a time series clustering method based on equivalence partitioning and morphological similarity.

In the field of machine learning, how many classes should be clustered in theory rather than relying on guesswork is a difficult problem that plagues the research and application of time series clustering methods. Based on four equivalence relationships that do not change the shape of time series data (i.e., horizontal translation, vertical translation, horizontal scaling, and vertical scaling), four equivalence partitioning methods for large-scale time series data sets are established, and algorithms for determining the number of clusters is given through quotient sets. These theoretical research results given in this paper provide a theoretical basis for how many clusters a large-scale time series should be clustered into.

With the help of the similarity metrics and their calculation formulas proposed in this paper, we can rigorously implement clustering of large-scale time series. The effectiveness of the proposed algorithms is verified by using the practical data in important industrial application scenarios as well as spacecraft monitoring scenarios.

It should be noted that although this study provides theoretical results on the number of clusters and computational metrics to measure morphological similarity between time series, morphological similarity is not a term with clear boundaries. How to accurately delineate similarity using metrics requires further research in the future.

## Data Availability

The datasets used and analyzed during the current study are available upon reasonable request from the corresponding author (S.H.). There are no restrictions on data availability.
